# Evaluating the effect of repetitive transcranial magnetic stimulation on sleep difficulties in children with autism spectrum disorder: a randomized controlled trial

**DOI:** 10.1093/sleepadvances/zpaf088

**Published:** 2025-12-05

**Authors:** Uchenna Ezedinma, Scott Burgess, Evan Jones, Jyoti Singh, Andrew Ladhams, Gary Campbell, Shauna Fjaagesund, Piotr Swierkowski, Adewuyi A Adeyinka, Alexandra P Metse, Terri Downer, Florin Oprescu

**Affiliations:** School of Health and Behavioral Science, University of the Sunshine Coast, 90 Sippy Downs Dr, Sippy Downs, QLD, 4556, Australia; Brain Treatment Center Australia, Ground Floor/19-31 Dickson Rd, Morayfield, QLD, 4506, Australia; The Thompson Institute, University of the Sunshine Coast, 12 Innovation Pkwy, Birtinya, QLD, 4575, Australia; Queensland Children's Lung and Sleep Specialists, 148 Logan Rd, Woolloongabba, QLD, 4102, Australia; School of Health and Behavioral Science, University of the Sunshine Coast, 90 Sippy Downs Dr, Sippy Downs, QLD, 4556, Australia; Brain Treatment Center Australia, Ground Floor/19-31 Dickson Rd, Morayfield, QLD, 4506, Australia; Health Hub Doctors Morayfield, Ground Floor/19-31 Dickson Rd, Morayfield, QLD, 4506, Australia; Brain Treatment Center Australia, Ground Floor/19-31 Dickson Rd, Morayfield, QLD, 4506, Australia; General Practice Unit, Faculty of Medicine, University of Queensland, 20 Weightman St, Herston, QLD, 4006, Australia; School of Health and Behavioral Science, University of the Sunshine Coast, 90 Sippy Downs Dr, Sippy Downs, QLD, 4556, Australia; Brain Treatment Center Australia, Ground Floor/19-31 Dickson Rd, Morayfield, QLD, 4506, Australia; School of Health and Behavioral Science, University of the Sunshine Coast, 90 Sippy Downs Dr, Sippy Downs, QLD, 4556, Australia; The Thompson Institute, University of the Sunshine Coast, 12 Innovation Pkwy, Birtinya, QLD, 4575, Australia; Health Hub Doctors Morayfield, Ground Floor/19-31 Dickson Rd, Morayfield, QLD, 4506, Australia; General Practice Unit, Faculty of Medicine, University of Queensland, 20 Weightman St, Herston, QLD, 4006, Australia; School of Health and Behavioral Science, University of the Sunshine Coast, 90 Sippy Downs Dr, Sippy Downs, QLD, 4556, Australia; Health Hub Doctors Morayfield, Ground Floor/19-31 Dickson Rd, Morayfield, QLD, 4506, Australia; Queensland Children's Lung and Sleep Specialists, 148 Logan Rd, Woolloongabba, QLD, 4102, Australia; Southern Cross University, Southern Cross Drive, Bilinga, QLD, 4225, Australia; School of Health and Behavioral Science, University of the Sunshine Coast, 90 Sippy Downs Dr, Sippy Downs, QLD, 4556, Australia; School of Psychological Science, University of Newcastle, University Drive, Callagan, NSW, 2308, Australia; School of Health and Behavioral Science, University of the Sunshine Coast, 90 Sippy Downs Dr, Sippy Downs, QLD, 4556, Australia; School of Health and Behavioral Science, University of the Sunshine Coast, 90 Sippy Downs Dr, Sippy Downs, QLD, 4556, Australia

**Keywords:** alpha rhythm, repetitive transcranial magnetic stimulation, autism spectrum disorder, sleep difficulties, polysomnography, children

## Abstract

**Study Objectives:**

Evaluate the effect and safety of alpha rhythm-guided repetitive transcranial magnetic stimulation (α-rTMS) on sleep difficulties in children with autism spectrum disorder (ASD).

**Methods:**

Twenty children (6–12 years old; 16 males; 4 females) with ASD level 2 were randomly assigned (1:1 ratio) to a treatment group (TG) or a waitlist control group (WLCG) (T1). The TG received ten α-rTMS sessions over two weeks, while the WLCG acted as control for that period (T2). Next, the WLCG received α-rTMS for two weeks (T3). All study participants were followed up at one (T4) and four (T5) months. Sleep difficulties were measured using the Children’s Sleep Habit Questionnaire (CSHQ), Actigraphy, and Polysomnography (PSG).

**Results:**

Group-by-time interactions indicated that the TG had greater improvements than the WLCG in total CSHQ score (*p=.008*) and, bedtime resistance (*p=.003*), sleep onset delay (*p=.004*), and sleep duration (*p*=.003) subdomain scores. When the WLCG received the α-rTMS, there were improvements in their sleep-disordered breathing (*p=.001*), parasomnia (*p=.*002) and sleep duration (*p=.018*) subdomain scores, while PSG data showed improved Waking After Sleep Onset (WASO) (*p=.014*), Sleep efficiency (*p=.046*), and N2 stage (*p=.039*). The improved CSHQ scores persisted, with actigraphy data showing significant improvement in WASO at T4 and T5. Side effects of α-rTMS were mild and transient.

**Conclusions:**

This RCT study presents preliminary evidence on the effect and safety of α-rTMS in improving subjective sleep difficulties in children with ASD, with effects lasting up to four months post-intervention. Further studies using a larger sample size and sham-controlled group are warranted.

**Clinical Trial Registration:**

The trial was registered on July 11, 2023 within the Australian New Zealand Clinical Trials Registry (ANZCTR) https://www.anzctr.org.au/TrialSearch.aspx with registration number: ACTRN12623000757617.

**Statement of Significance**

There is a need for new interventions that address the prevalent and significant sleep difficulties in children with autism spectrum disorder (ASD). This study presented preliminary evidence on the safety and effect of alpha rhythm-guided repetitive transcranial magnetic stimulation as a potential therapeutic option for improving sleep difficulties in children with ASD. Such technology-based intervention may provide an alternative approach to autistic children who do not respond to behavioral and or pharmacological-based sleep interventions. However, future studies using a larger sample size and sham-controlled design are warranted to translate this intervention into clinical practice.

## Introduction

Autism spectrum disorder (ASD) is a heterogeneous and complex neurodevelopmental disorder broadly characterized by core features of communication and social skills impairments and repetitive behavioral patterns [1]. Sleep difficulties are estimated to occur in 50%–80% of children with ASD compared to 11%–37% in typically developing children [2]. The frequently reported sleep difficulties include bedtime resistance, problems falling asleep and staying asleep, sleep fragmentation and early morning awakenings [2, 3]. These are associated with worsening core ASD features [4], as well as behaviors, cognition and development [5].

Consequently, the National Sleep Foundation identifies children with ASD as one of the highest-priority populations for sleep research [6]. Behavioral, medical, and neurobiological factors have been proposed as the mechanisms underlying sleep difficulties that co-occur in ASD [2]. One proposed neurobiological factor within the literature is an imbalance in excitatory and inhibitory neurotransmitters, such as the glutamate and GABAergic systems [7, 8]. Therefore, interventions that can improve these systems may have the potential to address sleep difficulties within the population [9].

An intervention that can target these systems is repetitive transcranial magnetic stimulation (rTMS). rTMS is a stimulation technique in which an electrical current passes through a coil positioned on the scalp, producing a magnetic field that alters neuronal activity at cortical and subcortical sites [10]. Low-frequency rTMS (<1 Hz) inhibits neuronal function, while high-frequency rTMS (>5 Hz) excites neuronal function [11]. These effects on neuronal functions may produce positive physiological and behavioral outcomes. Studies have summarized the potential outcomes of rTMS on core ASD features [12] and primary sleep difficulties [13].

Three studies have reported on rTMS involving children with ASD and sleep difficulties [14–16]. It has been hypothesized that the positive sleep outcomes may be underpinned by rTMS effects on the imbalanced excitatory and inhibitory neurotransmitter systems, such as the glutamate and GABAergic systems (Yan et al. 2024), as well as sensory abnormalities [15]. However, these studies lacked a control group, objective sleep measures and long-term follow-up [9, 14]. In addition, recent reviews have recommended a guided rTMS protocol for ASD [17] and sleep difficulties [13] to substantiate the evidence from current studies.

Gallop et al. [17] argued that a guided rTMS protocol, compared to standard rTMS, will account for ASD-specific or inter-individual variabilities in brain characteristics and any clinical heterogeneity, such as type and severity of co-occurring sleep difficulties. A lack of a neuro-navigated system that minimizes inter-and intra-individual variabilities was identified as a limitation of rTMS studies into primary sleep difficulties [13]. Experts have opined that a guided rTMS protocol is necessary for optimizing stimulation protocol and clinical outcomes [18]. Electroencephalographic data have been suggested for use in guiding rTMS protocol [13, 19].

The electroencephalogram (EEG) measures neuronal activity at cortical sites and is primarily defined by five rhythms: delta (0–4 Hz), theta (4–8 Hz), alpha (8–13 Hz), beta (13–25 Hz), and gamma (> 25 Hz). Alpha rhythm is the most salient eyes-closed, awake and relaxed EEG rhythm, a potential marker for neural development, cognition and behavioral functioning [20, 21]. It’s being proposed as an electrophysiological marker *for pineal gland melatonin secretion during sleep in individuals with autism* [22, 23]. Additionally, a randomized sham-controlled rTMS study on insomniacs found that the alpha rhythm could be a potential biomarker for predicting therapeutic outcomes [24].

Children with autism have a U-shaped EEG spectral profile, characterized by excessive power in delta, theta, beta, and gamma rhythms, suggesting reduced alpha rhythm power [25]. A recent systematic review also concluded that their alpha rhythms also bear reduced long-range functional connectivity, especially within the prefrontal cortex and other brain regions [26]. The *power* and *connectivity* of alpha rhythms have been closely linked to several physiological functions, including cognitive performance, working memory, attention, and social cognition [27, 28] as well as sleep in individuals with autism [29].

Thus, rTMS using a frequency within the alpha rhythm (8-13 Hz), i.e. high-frequency rTMS (>5 Hz), aims to excite neuronal functions [11] and guide the attempted restoration of the delayed alpha rhythm within the cortices of children with ASD. The rTMS frequency *can be theoretically set to 10 Hz* [15] or *algorithmically derived from the EEG of an autistic subject and then used to guide an individualized rTMS protocol* [16, 19]. The latter approach minimizes inter-and intra-individual variabilities [13]. For instance, a study showed that an individual’s alpha rhythm varied daily and these variabilities were related to their trait anxiety [30].

The algorithmically derived alpha rhythm used to guide rTMS frequency is informed by an appropriate and typical posterior-to-frontal alpha frequency gradient in individuals with autism [31, 32]. Alpha rhythm-guided rTMS (α-rTMS) has been reported to improve atypical alpha rhythms, autistic features [19] and sleep difficulties in children [15, 16]. However, these studies are sparse and limited by their small sample size, lack of control groups and objective sleep measures [9]. A control group, objective measures, and long-term follow-up are warranted for substantiating the clinical outcomes of α-rTMS [17].

Therefore, this RCT study aimed to evaluate the effects and report on the safety of α-rTMS on sleep difficulties in children with ASD using the Children’s Sleep Habit Questionnaire (CSHQ), Polysomnography (PSG) and Actigraphy (ACT).

The proposed study hypotheses were:

1. The treatment group would show significant improvements in sleep difficulties following α-rTMS compared to the waitlist control group (phase 1).
2. The waitlist control group would show significant improvements in sleep difficulties following the α-rTMS compared to the waitlist period (phase 2).
3. Following α-rTMS, its effect on the participant’s sleep difficulties would not significantly change at one (T4) and four (T5) months post-study follow-up (phase 3).

## Methods

### Study design, setting and procedure

**Figure 1:**
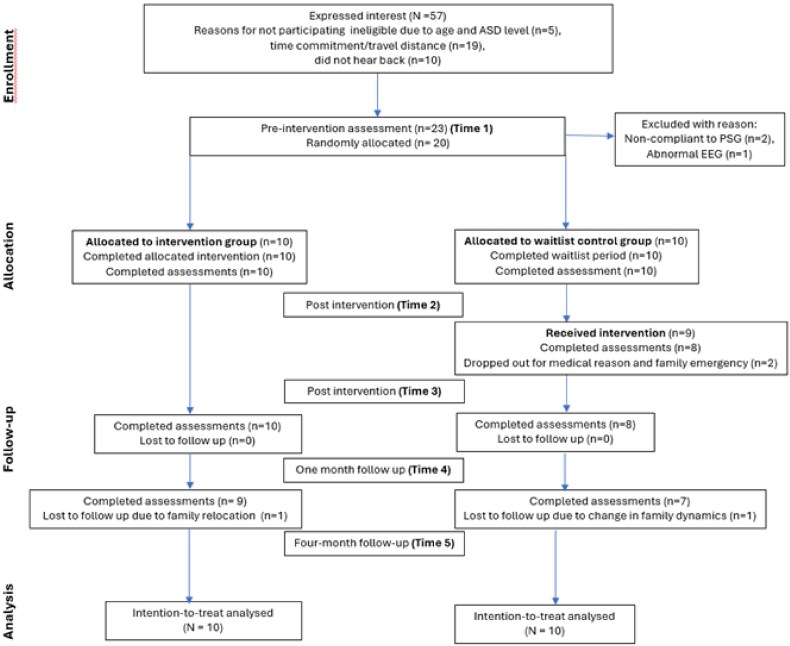
CONSORT diagram showing the flow of participants through the trial.

This research was designed as a randomized, waitlist-controlled, open-label trial. The study was conducted at no cost to participants within a private outpatient rTMS center in North Brisbane, Australia. Study phase 1 (T1 vs T2) included pre-intervention assessment (T1), randomization, intervention delivery to the treatment group (TG), while the waitlist control group (WLCG) acted as a control (waitlist period), followed by post-intervention assessment (T2). In study phase 2 (T2 vs T3), the intervention was delivered to WLCG and assessed immediately post-intervention (T3). Study phase 3 (T3 to T5) included assessments of both groups at one (T4) and four (T5) months post-study follow-up (Figure 1).

A waitlist-controlled group could be best suited for a small sample-sized study with limited resources [33] that involves testing a new intervention [34]. Offering the intervention to the waitlist participants after the trial period presents a more ethical approach [35] that encourages recruitment and reduces dropout rates [36, 37]. It also controls for threats to internal validity such as regression to the mean or spontaneous remission [38]. However, a waitlist-controlled group (an inactive control) does not minimize the potential for a placebo effect [39]. However, it may be argued that a placebo effect can be minimized by including objective measures.

The primary caregivers provided written informed consent for their child to participate in the research and held the knowledge that they could drop out anytime during the trial. The Ethics Committee of the University of the Sunshine Coast, Australia, reviewed and approved the protocol and informed consent forms in February 2023, with ethics number S221766. The trial was registered with the Australian New Zealand Clinical Trials Registry (ANZCTR) with registration number: ACTRN12623000757617.

### Participants

The participants were recruited from the community through advertisements on the notice boards of relevant institutions, health consumer websites, and social media support groups that targeted primary caregivers of children with ASD. A total of 20 children were randomly assigned (1:1) to either the TG or WLCG.

### Inclusion and exclusion criteria

The study inclusion criteria were children (6–12 years old) with a clinical diagnosis of ASD (level 2) and reported sleep difficulties. All participants had a current ASD (level 2) diagnosis by a paediatrician based on the Diagnostic and Statistical Manual of Mental Disorders, Fifth Edition (DSM-5) [1]. Reported sleep difficulties were based on the clinical cut-off of >41 on the Children’s Sleep Habits Questionnaire (CSHQ) as rated by the primary caregiver. The study excluded children with (1) poor compliance to polysomnography (PSG) as assessed by their hesitancy to participate and (2) EEG showing possible spike and/or slow wave rhythm.

### Randomization

Following the pre-intervention assessment, eligible participants were randomly assigned (1:1) to the TG and WLCG in four and six-block sizes [40]. A research member (not involved in participant recruitment) generated the random allocation sequence, concealed it using sealed and opaque envelopes and securely stored it until assignment. As this trial used a randomized waitlist-controlled open-label design with objective measures, the study investigators, children and primary caregivers were not blinded to the treatment.

### Intervention

Each participant underwent a ten-minute eyes-closed electroencephalogram (EEG) on a Deymed Truscan EEG system and a fitted 21-lead FlexiCAP with standardized 10-20 positioning. Short qEEG recordings of 5-10 minutes are suited for compliance purposes in children with ASD and have been shown to yield reliable and valid data [16, 41–45].

The EEG data were processed by a proprietary algorithm that calculated and identified the individual stimulation frequency from the regional alpha rhythm (8-13 Hz range) within the posterior-occipital region of interest (ROI) consisting of P3, P4, Pz, O1, and O2 electrodes, as the underlying region is where the highest alpha rhythm is observed [46]. The alpha peak activity was extracted as an average across the posterior electrodes. Other computation variables include a 50 Hz notch filter and a 1 Hz high pass during recording. Sampling rate of 200 Hz, bandpass filter setting of 2-20 Hz, linked-ear as reference, and FCz electrode as ground were used for offline analysis. Epochs containing artifacts or segments without alpha activity were not included in the offline alpha peak frequency estimation analysis. Fast Fourier Transform in Persyst Insight II was used to generate a power spectrum density plot as per Welch’s method, which turns time-domain EEG activity into a single spectral frequency plot for each electrode and provides the power of each measured frequency in the EEG method using 5.12-second epochs with 50% window overlap and 0.098 frequency resolution.

The stimulation sites, dorsomedial prefrontal cortex (dmPFC) and medial parietal cortex (mPC), were chosen as they are part of the six main areas of dysfunction in autism that can be identified using a quantitative electroencephalogram [47]. These sites also bear reduced alpha rhythm power and long-range functional connectivity in autistic individuals [16, 48–50]. These sites were also identified in primary sleep difficulties [13]. Each participant received ten alpha rhythm-guided repetitive transcranial magnetic stimulation (α-rTMS) sessions delivered during consecutive business days on a CF-B65 butterfly coil and Magpro R30 TMS stimulator (Magventure Inc., Denmark). The α-rTMS protocol was 5-second stimulation at 8.0–13.0 Hz range, delivered first to the Pz site and then to the Fpz site, with 28-second intervals between 32 trains and at 40% and 50% output intensity for each site, respectively (Table 1). The output intensities were based on the standard procedure of the private outpatient rTMS center for reducing potential side effects, as supported by previous studies [16, 48, 51]. A research technician delivered the intervention with the rTMS coil oriented perpendicular to the midline of the stimulation sites [52]. Therefore, each participant received an individualized stimulation frequency between 8.0–13 Hz for 5 seconds over 32 trains and at 40% and 50% within Pz and FPz sites, respectively. One α-rTMS session is approximately 40 minutes.

**Table 1:** Summary of stimulation parameters

Stimulation parameters	
Sites	**FPz and Pz**
Frequency	8–13 Hz
Trains (intertrain interval)	32 (28 seconds)
Output intensity	40%–50%

Relevance of the data is provided in boldface.

### Outcome measures

All sleep measures were completed simultaneously at each assessment time point, and the group changes from baseline through multiple time points were used to analyse outcomes of sleep difficulties (see Table 2 for the outcome measures used at each study time point).

1. *Children’s Sleep Habit Questionnaire (CSHQ)*

**Table 2:** Measures and study time points

	Time 1	Time 2	Time 3	Time 4	Time 5
**CSHQ**	+x	+x	x	+x	+x
**PSG**	+x	+x	x	+x	+x
**ACT**	+	+x	x	+x	+x

+, Treatment group; x, Waitlist Control group.

Relevance of the data is provided in boldface.

The CSHQ is a widely used and accepted primary caregiver-reported questionnaire that captures the frequently reported paediatric sleep difficulties with adequate internal consistency, specificity and sensitivity [53]. It consists of 41 questions sub-grouped into bedtime resistance, sleep onset delay, sleep duration, sleep anxiety, night waking, parasomnias, sleep-disordered breathing, and daytime sleepiness parameters. Using a Likert scoring system, a total CSHQ score > 41 indicates sleep difficulties.

1. *Polysomnography (PSG)*

Polysomnography (PSG) is considered the reference standard for paediatric sleep studies. It allows for a comprehensive assessment of sleep onset latency, total sleep time, sleep efficiency, night awakening, sleep architecture and sleep-related breathing. Given its demonstrated adequacy and cost-effectiveness, this study used an unattended home PSG (level 2) [54, 55].

The study adapted the administration of PSG as described by Russo et al. [54]. One night-PSG was administered using the Nox-A1 device at the participant’s home. The monitored signals included frontal electroencephalogram (EEG: FpZ, F4, and F3. With FpZ, right (M2) & left (M1) mastoid bone placement used as online reference and ground)), two electrooculograms (EOG: 2 cm out and 2 cm up of the right eye [E2], & 2 cm out and 2 cm down of the left eye [E1]), chin electromyogram (EMG: midline beneath the jawbone of the left [1] & right [2] chin), electrocardiogram, inductance plethysmography (RIP) of the chest and abdominal movement, pulse oximetry (Nonin 3150 with one of 2 age-appropriate-sized probes), and audio. Airflow was assessed using cRIP flow, a calculated signal derived from the respiratory bands.

The polysomnogram was manually scored by an accredited paediatric sleep scientist (JG) according to the guidelines of the American Academy of Sleep Medicine for sleep staging in 30-s epochs [56] and reviewed by a paediatric sleep physician (SB). The sleep scientist and physician were blinded to the randomized groups. The sleep parameters included total sleep time (TST), sleep onset latency (SOL), arousal index, relative proportions of REM, N1, N2, and N3 sleep, Waking After Sleep Onset (WASO), and sleep efficiency (SE).

1. *Actigraphy (ACT)*

Actigraphy was conducted using the MotionWare 8®- CamNtech MotionWare 1.1.20 set to Mode 1 with record light and a 30-second Epoch. The device was worn on the participant’s less dominant wrist five days before and five days after the α-rTMS. The primary caregivers were encouraged to take off the device during water-based activities like swimming and to press the marker button at bedtime and on waking. The sleep parameters included total sleep time (TST) and Waking After Sleep Onset (WASO).

### Side effects

As per standard procedure at the practice, the research assistant delivering the α-rTMS at each session enquired from the child and primary caregiver of any traditional rTMS side effects such as headache, hyperactivity/irritability, pain at the stimulation site or for any adverse events such as seizures. This approach to side effect reporting has been used in previous alpha rhythm-guided rTMS studies [16, 45].

### Statistical analysis

Demographic and baseline data, stimulation frequencies and reported side effects were analyzed using standard descriptive and Student *t-*test statistics. Analyses for study hypotheses were conducted on IBM SPSS, version 29.0, using a linear mixed models analysis (LMM). All analyses were conducted to ensure assumptions, such as normality, homogeneity of variances, and sphericity, were met. Greenhouse–Geisser (GG) estimates were employed where appropriate.

For study hypothesis 1, a random intercept model with group and time as fixed effects and subjects (participants) as random effects was used to assess TG (T1 and T2) versus WLC*G* (T1 and T2). Time (T1 and T2) and group (TG and WLC) were used as the within and between-subject variables, respectively. That is Outcome ~ Group*Time + (1|Subject). A significant group x time interaction indicated whether α-rTMS was effective in improving the dependent variables. For the actigraphy data, the TG’s pre-post α-rTMS (T1 and T2) were analyzed using the Student *t-*test.

For study hypothesis 2, the *WLCG* data were analyzed using LMM. A random intercept model with time as fixed effects and subjects (participants) as random effects to determine the main effects of time across all dependent variables. Time (T1, T2, and T3), within-subject variables, were selected as the repeated effects. That is, Outcome ~ Time + (1|Subject). Given the probability of Type 1 error due to multiple comparisons, the Bonferroni correction was used to correct alpha across timepoints. A significant effect of time indicated whether the α-rTMS was effective in improving the dependent variables. For the actigraphy data, the WLCG’s pre-post α-rTMS (T2 and T3) were analyzed using the Student *t-*test.

For study hypothesis 3, both TG and WLCG post-α-rTMS (T2 and T3) and one (T4) and four (T5) months post-study follow-up data were analyzed using the same approach as hypothesis 2 to determine the effect of time across all dependent variables. Time (T2 to T5), within-subject variables, were selected as the repeated effects. An insignificant change in the mean difference between time points (T2 to T5) indicated whether the α-rTMS effect persisted for the dependent variables.

Statistical significance was set at *p<.05*. A power calculation was not conducted as ten participants per group is assumed to be sufficient, given that it can provide a good estimate of study variance [57]. The effect size was measured as partial eta-squared (ηp^2^) with a small (*>*0.01 to *<*0.06), medium (*>*0.07 to *<*0.13) and large (*>*0.14) scale [58].

## Results

A total of 20 children (age range = 6–12 years, mean age *+* SD = 9.1 *+* 1.55) participated in the RCT between October 2023 and September 2024 (see Figure 1: CONSORT diagram showing the flow of participants through the trial.

Table 3 summarizes participant demographic data at baseline. The TG had seven boys and three girls, while the WLCG had nine boys and a girl; the overall ratio of boys to girls was 4:1. Participants had an average of 3 years following the ASD diagnosis. Multiple and concurrent sleep difficulties such as bedtime resistance, sleep anxiety, prolonged sleep onset latency, nighttime waking, early waking/short sleep and daytime tiredness/sleepiness were the most frequently reported.

Between 25%–50% of participants were co-diagnosed with attention deficit hyperactivity disorder, anxiety, and specific learning difficulties. The most used medications included stimulants (*n* = 11, 55%), α - 2a adrenergic agonist (*n* = 7, 35%), and melatonin (*n* = 7, 35%), while frequently accessed allied therapies were occupational (*n* = 15, 75%), counselor/psychotherapy/psychology (*n* = 12, 60%), speech (*n* = 7, 35%), and exercise physiology/physiotherapy (*n* = 5, 25%).

There were no significant differences in demographic and CSHQ data between groups at baseline (Table 3). There were also no significant differences between the group’s polysomnography variables at baseline, except for total sleep time, which was significantly longer (*p*=*.020*) in the TG (M = 572.8 mins, SD = 37.5) compared to the WLCG (M = 516.3 mins, SD = 53.2). None of the participants’ polysomnograms indicated a sleep disorder requiring medical attention, and were within the range of age-matched developing children [59].

**Figure 2:**
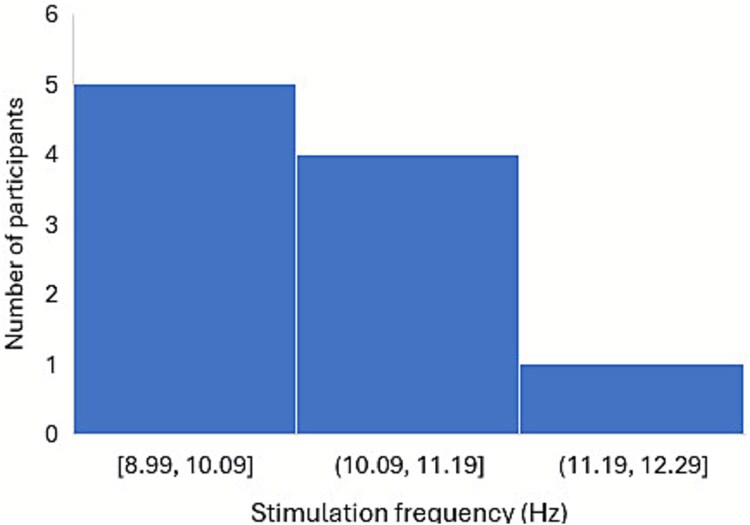
Histogram of the stimulation frequencies of TG participants.

**Figure 3:**
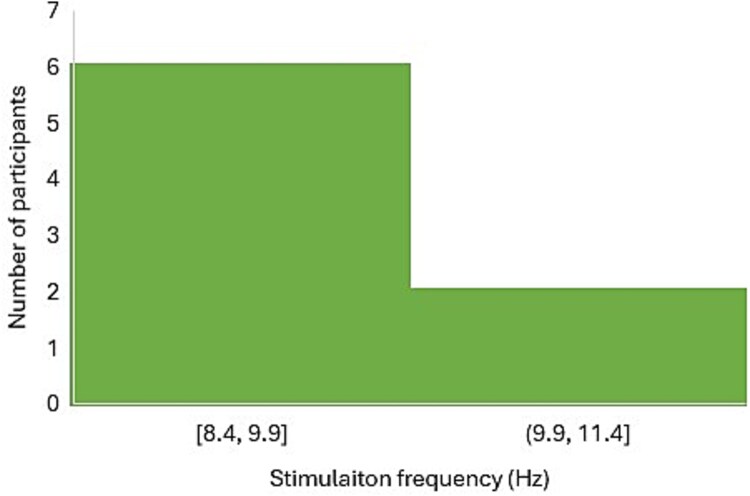
Histogram of the stimulation frequencies of WLCG participants.

Figures 2 and 3 show that half of the TG and WLCG participants were stimulated at the frontal and posterior regions using frequencies ranging from 8.99 to 11.2 Hz (M *+* SD = 10.10 *+* 0.6 Hz) and from 8.4 to 11.0 Hz (M = 9.54 Hz), respectively.

### Study hypothesis 1 was that the TG would show significant improvement in sleep difficulties following α-rTMS (T2 vs T1) compared to the WLCG (T2 vs T1).

**Figure 4:**
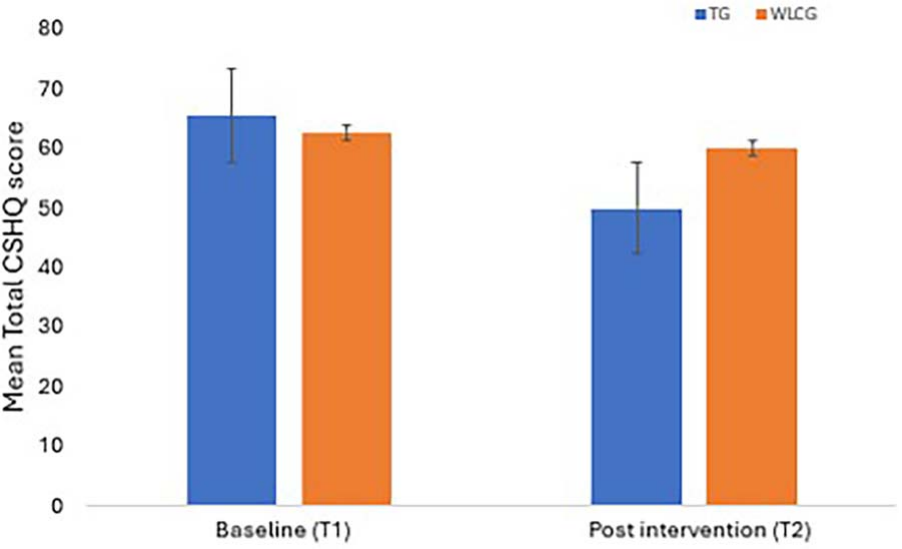
Mean Total Children’s sleep habit questionnaire (CSHQ) score–study 1.

CSHQ: The TG showed significantly improved sleep parameters compared to the WLCG. A significant group-by-time interaction was observed for the total CSHQ (*F* (1,18) = 8.71, *p=.008*, ES = 0.94), bedtime resistance (*F* (1,18) = 11.2, *p=.003*, ES = 0.92), sleep onset delay (*F* (1,18) = 10.87, *p=.004*, ES = 0.94) and sleep duration (*F* (1,18) = 11.8, *p=.003*, ES = 0.60) subdomain scores, indicating that α-rTMS effect on the TG changed significantly over time. The effect sizes were large (Table 4). The mean differences at T2 showed the TG significantly improved in total CSHQ (M = –9.8, *p=.013*), bedtime resistance (M = –2.9, *p*=.029), sleep onset delay (M = –0.8, *p=.014*), and sleep-disordered breathing (M = –0.7, *p=.010*) subdomain scores compared to the WLCG (Table 4). Figure 4 shows the trend towards decreasing Total CSHQ score in the TG.

**Table 3:** Participants’ demographic characteristics and baseline assessment

Baseline characteristics	TG (*n* = 10)	WLC (*n* = 10)	*Comparison*
*Demographics*			
Mean age (SD)/range	9 (1.49)/ 7–12	9.2 (1.68)/ 6–11	*p=.780*
Boys (*n)*	7 (70%)	9 (90%)	
Girls *(n)*	3 (30%)	1 (10%)	
Years since ASD diagnosis (SD)/range	2.9 (1.79)/1–6	3 (1.82)/1–6	*p=.900*
*Reported sleep difficulties*			
Bedtime resistance	7	6	
Sleep anxiety	5	3	
Prolonged sleep onset latency	7	4	
Nighttime waking	4	4	
Early waking/short sleep	6	5	
Daytime tiredness/sleepiness	3	3	
*Other comorbidities*			
Attention Deficit Hyperactivity Disorder	7	7	
Anxiety (General anxiety disorder, social anxiety)	6	4	
Dyslexia/ Dyscalculia/ Dysgraphia/sensory disorder	3	2	
*Existing medications*			
Yes	8	6	
No	2	4	
α-2a adrenergic agonist (clonidine, guanfacine)	5	2	
Melatonin	4	3	
Stimulant (Lisdexamfetamine, methylphenidate)	6	5	
*Existing allied health therapies*			
Counselor/psychotherapy/psychology	8	4	
Exercise physiology/physiotherapy	4	1	
Occupational therapy	10	5	
Speech therapy	4	3	
*Baseline primary caregivers-rated CSHQ*			
Mean CSHQ (SD)	65.3 (11.8)	62.5 (9.3)	*p=.560*
*Baseline home polysomnography assessment*			
	TG (*n* = 9^*^)	WLCG (*n* = 10)	
Mean total sleep time (SD)	572.8 (37.5)	516.3 (53.2)	***p=.020***
Mean sleep latency (SD)	37.8 (48.4)	33.8 (19.8)	*p=.820*
Mean WASO (SD)	34.1 (23.9)	57.5 (39.9)	*p*=*.130*
Mean sleep efficiency (SD)	88.6 (8.3)	83.8 (8.7)	*p=.180*
Mean S1 (SD)	1.2 (0.5)	1.7 (1.7)	*p=.370*
Mean S2 (SD)	37.9 (3.8)	33.9 (6.5)	*p=.120*
Mean S3 (SD)	33.4 (5.1)	36.2 (7.5)	*p=.350*
Mean REM (SD)	27.5 (4.1)	28.2 (5.2)	*p=.750*
Mean arousal index (SD)	7.2 (1.4)	6.3 (1.9)	*p=.270*

^*^ *One participant’s data was of low quality (i.e. didn’t have > 6 hr sleep with key channels present for at least 90% of the time).*

Home PSG: The TG showed insignificant improvements in PSG parameters compared to the WLCG. An insignificant group-by-time interaction was observed across all polysomnographic variables, indicating that the α-rTMS effect on the TG did not change significantly over time. The mean difference at T2 showed that the TG did not significantly improve across all PSG parameters compared to the WLCG (Table 5).

Actigraphy data: The TG showed worsening of sleep parameters post-α-rTMS. Table 6 showed a decrease in the mean total sleep time (T1 to T2: 465.5 to 452.7) and an increase in WASO (T1 to T2: 70.2 to 87.1) scores. However, the mean differences in both variables were insignificant.

### Study hypothesis 2 was that the WLCG would show significant improvements in sleep difficulties following α-rTMS (T3 vs T2) compared to the waitlist period (T2 vs T1).

**Figure 5:**
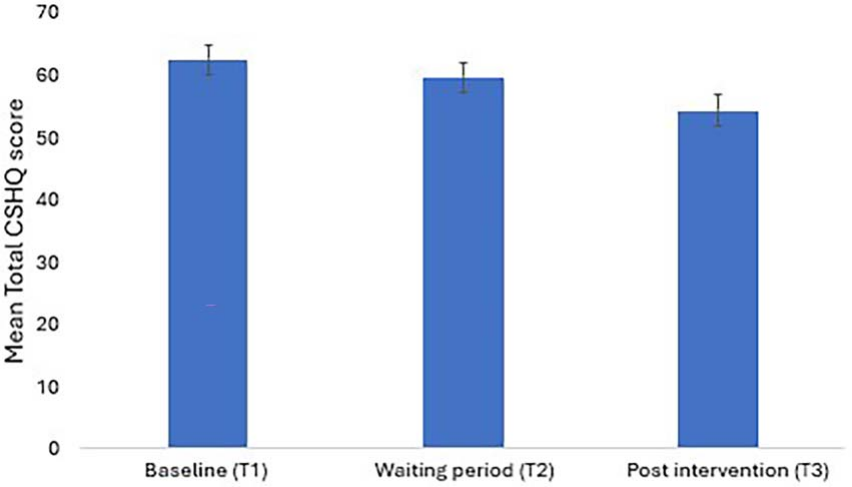
Mean Total Children’s sleep habit questionnaire (CSHQ) score–study 2.

CSHQ: The WLCG showed significantly improved sleep parameters following α-rTMS compared to the waitlist period. The main effect of time was significant for sleep-disordered breathing (*F* (1, 9) = 17.24, *p=.001*, ES = 0.99), parasomnia (*F* (1,9) = 7.63, *p=.012*, ES = 0.83) and sleep duration (*F* (1, 9) = 6.32, *p=.018*, ES = 0.03), subdomain scores. The effect sizes were small to large. The mean differences at T3 vs T2 were significant for sleep-disordered breathing (M = –0.9, *p=.008*) and parasomnia (M = –1.3, *p=.050*) subdomain scores (Table 7). Figure 5 shows the trend towards decreasing Total CSHQ score in the WLCG.

**Table 4:** Children’s Sleep Habit Questionnaire (CSHQ)–study 1

	TG		WLCG		Mean difference (*p-*value) at T2	Group^*^time interaction		
	T1 M (SE)	T2 M (SE)	T1 M (SE)	T2 M (SE)		F (df = 1, 18)	*p-value*	Effect size
**Bedtime resistance**	12.4 (0.9)	9.1 (0.9)	11.6 (0.9)	12.0 (0.9)	−2.9 (**0.029**)	11.20	**0.003**	0.92
**Sleep onset delay**	2.8 (0.1)	1.6 (0.2)	2.6 (0.1)	2.4 (0.2)	−0.8 (**0.014**)	10.87	**0.004**	0.94
Sleep anxiety	8.2 (0.8)	7.0 (0.7)	7.9 (0.8)	8.4 (0.7)	−1.4 (0.190)	2.77	0.105	0.81
Sleep-disordered breathing	3.9 (0.4)	3.3 (0.2)	5.1 (0.4)	4.0 (0.2)	−0.7 (**0.010**)	0.92	0.350	0.93
Parasomnia	11.0 (0.8)	8.7 (0.7)	10.5 (0.8)	10.1 (0.7)	−1.4 (0.166)	2.02	0.170	0.81
Night waking	6.3 (0.6)	4.3 (0.5)	5.5 (0.6)	4.9 (0.5)	−0.6 (0.402)	3.14	0.092	0.60
**Sleep duration**	7.3 (0.5)	4.9 (0.5)	6.2 (0.5)	5.5 (0.5)	−0.6 (0.438)	11.80	**0.003**	0.60
Daytime sleepiness	15.9 (1.5)	12.6 (1.2)	14.8 (1.5)	14.9 (1.2)	−2.3 (0.243)	2.77	0.112	0.79
**Total**	65.3 (3.8)	49.8 (2.6)	62.5 (3.1)	59.6 (2.3)	−9.8 (**0.013**)	8.71	**0.008**	0.94

Relevance of the data is provided in boldface.

**Table 5:** Home (level 2) Polysomnography – study 1

	TG		WLCG		Mean difference (*p-value*) at T2	Group^*^time interaction		
	T1 M (SE)	T2 M (SE)	T1 M (SE)	T2 M (SE)		F (df = 1, 18)	*p-value*	Effect size
Total Sleep Time (mins)	570 (14.5)	574.3 (15.5)	516.3 (13.8)	534.4 (14.9)	39.9 (0.078)	0.47	0.500	0.88
Sleep latency (mins)	36.4 (11.4	49.9 (8.4)	33.8 (10.8)	32.7 (8.0)	17.2 (0.157)	0.47	0.501	0.82
WASO	33.6 (10.5)	30.5 (6.4)	57.5 (9.9)	26.1 (6.1)	4.4 (0.624)	0.63	0.072	0.34
Sleep efficiency (%)	88.9 (2.7)	86.9 (1.6)	83.8 (2.6)	89.6 (1.5)	−0.8 (0.734)	3.91	0.062	0.76
N1 (%)	1.2 (0.4)	1.3 (0.2)	1.7 (0.4)	1.5 (0.2)	−0.2 (0.422)	0.17	0.687	0.51
N2 (%)	38.5 (1.7)	39.6 (2.0)	33.9 (1.8)	37.7 (1.9)	1.9 (0.489)	0.98	0.335	0.50
N3 (%)	34.78 (1.3)	30.9 (1.3)	36.2 (1.8)	32.8 1.3)	−1.9 (0.317)	0.03	0.856	0.69
REM (%)	26.8 (1.37	27.8 (1.3)	28.2 (1.5)	27.9 (1.3)	−0. 2 (0.916)	0.20	0.658	0.01
Arousal index (/hr)	7.2 (0.5)	5.9 (0.6)	6.3 (0.5)	5.8 (0.6)	−0.09 (0.922)	0.91	0.344	0.03

**Table 6:** ACT data for pre-post intervention (Treatment group, *n* = 6^*^)

	T1 Mean (SD)	T2 Mean (SD)	Mean difference	*p-value*	*Effect size*
Total sleep time (mins)	465.5 (42.9)	452.7 (37.4)	−12.8	0.546	0.10
WASO	70.2 (21.6)	87.1 (26.7)	16.9	0.119	0.34

^*^ *Four participants had lost data (i.e. didn’t wear the ACT device on one or more nights at T2).*

**Table 7:** Children’s Sleep Habit Questionnaire (CSHQ)–Phase 2

	Mean score			Mean difference			Effect of time		
	T1 M (SE)	T2 M (SE)	T3 M (SE)	T2–T1 *(p-value)*	T3–T1 *(p-value)*	T3–T2 *(p-value)*	*F* *(df = 1, 9)*	*p-value*	Effect size
Bedtime resistance	11.6 0.9)	12.0 (1.0)	11.9 (0.8)	0.4 (1.000)	0.3 (1.000)	−0.1 (1.000)	0.43	0.673	0.01
Sleep onset delay	2.6 (0.2)	2.4 (0.2)	2.0 (0.3)	−0.2 (1.000)	−0.6 (0.218)	−0.4 (0.794)	2.14	0.177	0.73
Sleep anxiety	7.9 (0.1)	8.4 (0.8)	8.3 (0.5)	0.5 (1.000)	0.5 (1.000)	−0.1 (1.000)	0.39	0.689	0.02
**Sleep-disordered breathing** ^*^	5.1 (0.4)	4.0 (0.1)	3.2 (0.1)	−1.1 (**0.036**)	−1.9 (**0.002**)	−0.9 (**0.008**)	17.24	**0.001**	0.99
**Parasomnia**	10.5 (0.7)	10.1(0.6)	8.7 (0.7)	−0.4 (0.729)	−1.7 (**0.012**)	−1.3 (**0.050**)	7.63	**0.012**	0.83
Night waking	5.5 (0.6)	4.9 (0.6)	4.9 (0.6)	−0.6 (0.576)	−0.5 (1.000)	0.0(1.000)	1.41	0.292	0.00
**Sleep duration** ^*^	6.2 (0.6)	5.5 (0.6)	5.4 (0.5)	−0.7 (**0.018**)	−0.8 (0.692)	−0.1 (1.000)	6.32	**0.018**	0.03
Daytime sleepiness	14.8 (1.3)	14.9 (1.5)	12.8 (1.2)	0.1 (1.000)	−1.9 (1.000)	−2.1 (0.976)	0.55	0.593	0.71
Total	62.5 (2.8)	59.6 (3.2)	54.3 (1.9)	−2.9 (0.445)	−8.1 (0.192)	−5.2 (0.777)	3.64	0.071	0.80

^*^Greenhouse–Geisser correction.

Relevance of the data is provided in boldface.

Home PSG: The WLCG showed an insignificant improvement in sleep parameters following α-rTMS compared to the waitlist period. The effect of time was significant for WASO (*F* (1,9) = 7.295, *p=.014*, ES = 0.60), Sleep efficiency (*F* (1,9) = 2.504, *p=.046*, ES = 0.51), and N2 sleep (*F* (1,9) = 4.596, *p=.039*, ES = 0.46). The effect size was large. The mean differences at T3 vs T2 were insignificant across all PSG variables. (Table 8).

Actigraphy data: The WLCG showed improved sleep parameters post α-rTMS. Table 9 showed an increase in the WLCG mean total sleep time (T2 to T3: 473.6 to 505.0) and a decrease in WASO (T2 to T3: 83.7.2 to 70.1) scores. However, the mean differences in both variables were insignificant.

### Study hypothesis 3 was that following α-rTMS (T2/T3), the effect on the participant’s sleep difficulties would not significantly change at one (T4) and four (T5) months post-study follow-up.

**Figure 6:**
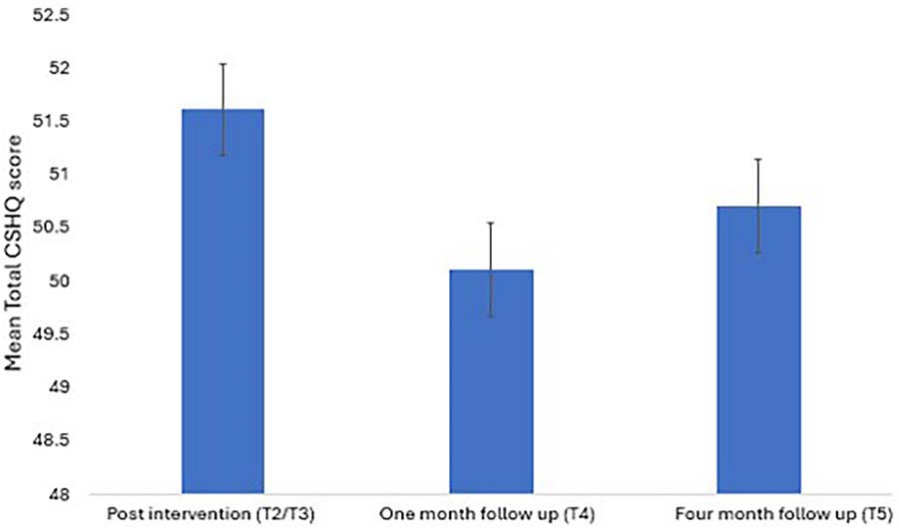
Mean Total Children’s sleep habit questionnaire (CSHQ) score–study 3.

CSHQ: Following α-rTMS (T2/T3), the effect on the participant’s sleep difficulties did not significantly change at T4 and T5, except for sleep duration subdomains. The effect of time was significant for the sleep duration subdomain (*F* (1, 15) = 3.5, *p=.050*, ES = 0.80). The effect size was large. Specifically, for subdomains that significantly improved post-α-rTMS (T2/T3), such as total CSHQ score (T4 vs T2/T3: –1.4, T5-T2/T3: -0.9), bedtime resistance (T4 vs T2/T3: –0.7, T5-T2/T3: -0.7), sleep onset delay (T4 vs T2/T3: 0.0, T5-T2/T3: 0.0), sleep-disordered breathing (T4 vs T2/T3: 0.2, T5-T2/T3: 0.1), and parasomnia (T4 vs T2/T3: –0.5, T5-T2/T3: 0.1), the mean difference at T4 and T5 were insignificant (Table 10). Figure 6 shows persistence in the Total CSHQ score across timepoints.

**Table 8:** Home (level 2) Polysomnography–Study 2

	Mean value			Mean difference			Effect of time		
	T1 M (SE)	T2 M (SE)	T3 M (SE)	T2–T1 *(p-value)*	T3– T1 *(p-value)*	T3– T2 *(p-value)*	*F* *(df = 1, 9)*	*p-value*	Effect size
Total sleep time (mins)	516.3 (15.9)	534.4 (18.2)	551.8 (23.8)	18.1(0.500)	35.5 (0.715)	17.2 (1.000)	2.050	0.194	0.42
**WASO**	57.5 (11.9)	26.1 (3.1)	38.2 (14.9)	−31.5 **(0.048**)	−19.3 (1.000)	12.1 (1.000)	7.295	**0.014**	0.60
Sleep latency (mins)	33.8 (5.9)	32.7 (4.7)	30.1 (5.6)	−1.1 (1.000)	−3.7 (1.000)	−2.6 (1.000)	0.148	0.867	0.21
**Sleep efficiency (%)**	83.8 (2.6)	89.6 (1.2)	87.6 (2.7)	5.8 (**0.049)**	3.8 (0.937)	−1.9 (1.000)	2.504	**0.046**	0.51
N1 (%)	1.7 (0.5)	1.5 (0.3)	1.7 (0.3)	−0.2 (1.000)	0.01 (1.000)	0.2 (1.000)	0.082	0.922	0.32
**N2 (%)**	33.9 (1.9)	37.7 (1.6)	39.1 (1.5)	3.8 (0.137)	5.189 (**0.036**)	1.4 (0.838)	4.596	**0.039**	0.46
N3 (%)	36.2 (2.2)	32.8 (1.2)	33.1 (1.9)	−3.4 (0.263)	−3.1 (0.118)	0.3 (1.000)	3.512	0.072	0.04
REM (%)	28.2 (1.6)	27.9 (1.3)	25.8 (2.1)	−0.2 (1.000)	−2.5 (0.495)	−2.2 (0.860)	1.235	0.334	0.62
Arousal index (/hr)	6.3 (0.6)	5.8 (0.7)	6.3 (0.6)	−0.5 (0.773)	−012 (1.000)	0.4 (1.000)	1.122	0.365	0.38

Relevance of the data is provided in boldface.

**Table 9:** ACT data for pre-post intervention (Waitlist control group, *n* = 8)

	T2 mean (SD)	T3 mean (SD)	Mean difference	*p-value*	*Effect size*
Total sleep time (mins)	473.6 (34.2)	505.0 (96.9)	31.4	0.443	0.17
WASO	83.7 (32.5)	70.1 (39.4)	−13.6	0.336	0.13

^*^ *Two participants had lost data (i.e. didn’t wear the ACT device on one or more nights at T2).*

**Table 10:** Children’s Sleep Habit Questionnaire (CSHQ)–Study 3

	Mean value			Mean difference		Effect of Time			
	T2/T3 M (SE)	T4 M (SE)	T5 M (SE)	T4– T2/T3 *(p-value)*	T5– T2/T3 *(p-value)*	T5–T4 *(p-value)*	*F* *(df = 1, 15)*	*p-value*	Effect size
Bedtime resistance	10.6 (0.6)	9.8 (0.6)	9.8 (0.6)	−0.7 (1.000)	−0.7 (0.877)	0.0 (1.000)	0.85	0.446	0.00
Sleep onset delay	1.8 (0.2)	1.8 (0.1)	1.8 (0.2)	0.0 (1.000)	0.0 (1.000)	0.0 (1.000)	0.01	0.990	0.00
Sleep anxiety	7.6 (0.6)	7.1 (0.6)	7.3 (0.5)	−0.5 (1.000)	−0.3 (1.000)	0.2 (1.000)	0.43	0.660	0.12
Sleep-disordered breathing	3.2 (0.1)	3.5 (0.2)	3.3 (0.2)	0.2 (0.286)	0.1 (1.000)	−0.1 (1.000)	1.68	0.234	0.51
Parasomnia	8.8 (0.5)	8.3 (0.5)	8.9 (0.5)	−0.5 (1.000)	0.1 (1.000)	0.6 (0.330)	1.68	0.215	0.60
Night waking	4.5 (0.4)	4.5 (0.4)	4.3 (0.3)	0.0 (1.000)	−0.2 (1.000)	−0.2 (1.000)	0.38	0.691	0.24
**Sleep duration**	5.1 (0.3)	4.8 (0.4)	4.1 (0.3)	−0.3 (1.000)	−0.9 (0.061)	−0.7 (0.186)	3.50	**0.050**	0.80
Daytime sleepiness	12.6 (0.8)	12.3 (0.8)	13.3 (0.9)	−0.3 (1.000)	0.6 (1000)	0.9 (0.631)	0.61	0.555	0.59
Total CSHQ	51.6 (1.4)	50.1(1.7)	50.7 (1.8)	−1.4 (0.857)	−0.9 (1.000)	0.6 (1.000)	0.68	0.520	0.11

Relevance of the data is provided in boldface.

Home PSG: The PSG parameter following α-rTMS (T2/T3) did not significantly change at one- and four-month post-study follow-ups. The effect of time was insignificant across sleep parameters. The mean difference between T2/T3 vs T4 and T2/T3 vs T5 was insignificant for all sleep parameters (Table 11).

Actigraphy data: There was a significant change in the participant’s sleep parameter at T4 and T5. The effect of time was significant for total sleep time (*F* [1, 17] = 3.6, *p=.048,* ES = 0.87) and WASO (*F* [1, 17] = 6.87, *p=.009*, ES = 0.39). Specifically, the mean difference of WASO between T4 vs T2/T3 (M = –24.1, *p=.*009) and T5 vs T2/T3 (M = –28.8, *p=.*008) was significant. The effect sizes were large (Table 12).

#### Side effects

Reports from the child and primary caregiver on side effects and α-rTMS day(s) they occurred were: headaches (*n* = 10, 58%; on α-rTMS days 1-3rd, 6th, and 7th), irritability/agitation/hyperactivity/emotional (*n* = 7, 41%: on α-rTMS days 1st–8th), discomfort at stimulation site (*n* = 5, 29%; on α-rTMS days 1st, 3rd–5th), unusually tired and quiet (*n* = 3, 17%; on α-rTMS days 2nd, 7th, and 8th) and dizziness and nausea (*n* = 2, 12%; on α-rTMS days 3rd, 4th, 5th, and 8th). No adverse events, such as seizures, were reported.

## Discussion

The study found that α-rTMS was well-tolerated and associated with improved subjective reports of sleep difficulties in children with ASD, with effects lasting up to four months post-intervention.

α-rTMS significantly improved the participant’s total CSHQ scores and several subdomains, such as bedtime resistance, sleep onset delay, sleep-disordered breathing (SDB), parasomnia, and sleep duration. The improved sleep difficulties are clinically relevant as they are the most frequently reported in autistic children [2, 3]. In fact, the significant improvement in sleep duration was consistent across study hypotheses, suggesting that a frequency range of 8.4–11.2 Hz rTMS delivered to the frontal and posterior regions could be a potential therapeutic option for total sleep time in children with autism.

**Table 11:** Home (level 2) Polysomnography–Study 3

	Mean value				Mean difference		Effect of time		
	T2/T3 M (SE)	T4 M (SE)	T5 M (SE)	T4– T2/T3 *(p-value)*	T5– T2/T3 *(p-value)*	T5– T4 *(p-value)*	*F* *(df = 1, 16)*	*p-value*	Effect size
Total sleep time (mins)	565.5 (12.6)	548.6 (16.7)	576.5 (12.1)	−16.9 (1.000)	10.9 (1.000)	27.9 (0.974)	0.59	0.564	0.79
Sleep latency (mins)	40.2 (6.9)	59.9 (21.5)	29.9 (8.7)	19.7 (1.000)	−10.3 (1.000)	−29.9 (0.810)	0.66	0.529	0.78
WASO	37.1 (8.4)	30.4 (3.5)	24.1 (3.5)	−6.7 (1.000)	−12.9 (0.591)	−6.3 (0.693)	1.38	0.287	0.77
Sleep efficiency (%)	87.6 (1.7)	85.8 (2.7)	91.5 (1.5)	−1.7 (1.000)	3.9 (0.219)	5.6 (0.454)	2.14	0.157	0.88
N1 (%)	1.5 (0.2)	1.4 (0.2)	1.2 (0.2)	−0.1 (1.000)	−0.3 (0.836)	−0.2 (1.000)	0.70	0.512	0.51
N2 (%)	39.6 (1.5)	37.7 (1.8)	36.4 (1.9)	−1.9 (0.912)	−3.2 (0.455)	−1.2 (1.000)	1.24	0.316	0.34
N3 (%)	32.5 (1.3)	33.3 (1.3)	34.4 (2.0)	1.0 (1.000)	2.2 (0.506)	1.1 (1.000)	1.22	0.354	0.31
REM (%)	26.7 (1.3)	28.1 (1.1)	27.6 (1.3)	1.4 (1.000)	0.9 (1.000)	−0.5 (1.000)	0.45	0.648	0.15
Arousal Index (/hr)	6.2 (0.4)	7.0 (0.5)	7.0 (0.6)	0.8 (0.639)	0.8 (0.446)	0.0 (1.000)	1.31	0.302	0.00

**Table 12:** ACT data–Study 3

	Mean value			Mean difference					
	T2/T3 M (SE)	T4 M (SE)	T5 M (SE)	T4 - T2/T3 *(p-value)*	T5 - T2/T3 *(p-value)*	T5 - T4 *(p-value)*	*F* *(df = 1, 17)*	*p-value*	Effect size
**Total sleep time (min)**	472.6 (16.8)	466.6 (11.3)	502.2 (16.7)	−5.95 (1.000)	29.58 (0.634)	35.53 (**0.050**)	3.62	**0.048**	0.87
**WASO**	81.01 (7.5)	56.83 (5.0)	52.21 (6.5)	−24.1 (**0.009**)	−28.8 (**0.008)**	−4.6 (0.923)	6.87	**0.009**	0.39

Relevance of the data is provided in boldface.

It could be proposed that α-rTMS improved these sleep difficulties by correcting the putative excitatory/inhibitory neurotransmitter imbalance via normalizing the over-excitatory glutamatergic pathway and consequent promotion of the under-inhibitory GABAergic pathway [14, 60]. In turn, the improved under-inhibitory GABAergic pathway promotes the “*idling*” state of alpha rhythms, which has been linked to the active inhibition of irrelevant sensory responses [25]. The improved sensory abnormalities have been thought to partially mediate sleep difficulties in children with autism [15].

Furthermore, the changes in alpha rhythms used in guiding the rTMS protocol may serve as a potential biomarker for the observed sleep outcomes [24]. As reported elsewhere, participants’ alpha rhythms demonstrated improved alpha rhythms, especially within the TG’s frontal region [61]. This biomarker, i.e. increased alpha rhythm, may underpin the significant improvement in more sleep subdomains compared to the WLCG following α-rTMS. In addition, methylenetetrahydrofolate reductase gene variant status within the WLCG may have impacted the changes to their alpha rhythms [61].

However, evidence supporting this proposed mechanism remains not fully established [13] or applicable across all sleep difficulties. For instance, in the improvement of SDB, the proposed positive effect of rTMS on cortical excitability or upper airway muscles, which enhances airflow dynamics without arousing patients [60, 62], is considered inconsistent and inconclusive [63]. Such inconsistencies may be due to a lack of objective sleep measures such as polysomnography (PSG).

Further, these inconsistencies may arise from the discordance between sleep measurement tools [64]. For instance, in study phase 2, PSG data showed mild improvement in the mean total sleep time compared to a clinically relevant increase of over 30 minutes from the actigraphy tool [65]. Such inconsistencies may be due to a more limited PSG study time (one night) compared to the actigraphy that is administered over multiple sleep nights per assessment period. Moreso, the discordance between objective and subjective sleep measurement tools have also been reported [64].

Notwithstanding, there was a general concordance in the sleep trends as measured by both the objective and subjective tools at one and four months post-α-rTMS follow-up. The CSHQ showed significantly improved sleep duration subdomain over time. This trend may also have been observed in actigraphy, as there were significant improvements in WASO and total sleep time. Thus, as the primary caregiver reported the child slept longer, there may have been concordance with the actigraphy, which showed longer sleep time and reduced WASO. However, no significant improvement was observed in the PSG data, likely due to the limited number of nights.

The persistence of the α-rTMS effect up to four months post-intervention is not without precedent, as a recent systematic review concluded that such an effect on sleep difficulties could persist for *weeks* [13]. Such a persisting effect should be considered within the context of the reported side effects. This study reported side effects as mild and transient, and not occurring beyond the 8^th^ session of α-rTMS. Overall, the lack of adverse events, such as seizures, adds to recent evidence that reports high-frequency (>5 Hz) rTMS to be safe in children with ASD [16, 45, 66].

Although not the primary aim of this study, it is worth noting that social skills and the quality of life of the child and their primary caregiver were secondary outcomes assessed, as documented in the trial registration. Results showed significant improvements in the quality of life of the children and their primary caregivers at different assessment time points; these findings may support the effects and safety of α-rTMS. However, there was no significant improvement in the children’s social skills, which was attributed to the small number of α-rTMS sessions (For a full read, see Ezedinma et al. [67]).

### Study strengths and limitations

To the author’s knowledge, this is the first randomized controlled trial study on the alpha rhythm-guided rTMS (α-rTMS) protocol among children with ASD and sleep difficulties. The recruitment of participants between 6 and 12 years old and ASD level 2 improved the homogeneity within the sample, thus strengthening the internal validity of the findings within the cohort. The participant’s male-to-female ratio of 4:1 is comparable to that reported in epidemiological studies [68, 69]. This gender distribution suggests that the study population is representative of the broader ASD population.

Furthermore, the α-rTMS effects were observed in the treatment and waitlist control groups, thereby accepting the study’s hypotheses. The improved sleep difficulties were predominantly those frequently reported within the community [70]. The combination of objective and subjective sleep measures is novel, which allowed for a robust assessment of α-rTMS effects. Specifically, this study is the first to utilize home (level 2) polysomnography within an interventional RCT trial to assess for sleep disorders, despite reported concerns of heightened sensory sensitivities and low tolerance within the population [15].

The study results should be considered within the context of several limitations. Although the sample size gives a reasonable estimate of variance [57], it limited the use of age, sex, comorbidities such as ADHD [71], interindividual EEG variation due to methylenetetrahydrofolate reductase mutation [72] and time of electroencephalogram collection [73] as covariates in the analysis of study outcomes. Other confounders, such as the impact of gender and ADHD on total sleep time between groups, were not accounted for [71, 74].

As an open-label RCT study, there is potential for a placebo effect or response bias impacting the subjective assessment of study outcomes. Moreover, the study lacked electrophysiological measures for sensory abnormalities, such as electrodermal or skin conductance levels [75, 76]. These electrophysiological tools may provide an objective assessment of the key mediators of sleep difficulties, such as tactile sensitivity, within the population [15, 77].

Due to the study inclusion criteria, the external validity of the study outcomes may only apply to male children (6-12 years old) with ASD level 2 and sleep difficulties. There should be caution in attributing the improved sleep difficulties to the α-rTMS effect, given that most participants’ concurrent use of medical and behavioral therapies may have had an additive or complementary effect on the outcomes. This study employed a limited number of α-rTMS sessions; more rTMS sessions have been hypothesized to yield durable and long-lasting clinical outcomes [41, 42].

### Recommendations

The preliminary evidence from this study supports the recommendation for further large sample-sized RCTs. A large, randomized, sham-controlled, double-masked study with a large sample size is required to accurately estimate the efficacy of a higher number α-rTMS sessions within a more heterogeneous population. In the sham α-rTMS design, the control group should have the same coil orientation and mimic the stimulation sound as the treatment group to appropriately mask the treatment technician and primary caregiver.

To provide an accurate sleep measure on objective tools, systematic desensitization [78] or two consecutive nights of home PSG studies [79, 80] or a single night of home PSG with video monitoring is recommended alongside multiple nights of actigraphy study. It was observed that the analyzed EEG of participants following α-rTMS [61] may align with the sleep outcomes in this study. Therefore, future studies using EEGs to predict the rTMS effect on sleep difficulties should be investigated [24].

## Conclusions

This RCT study presents preliminary evidence on the effect and safety of α-rTMS in improving subjective sleep difficulties in children with ASD, with effects lasting up to four months after the intervention. Future studies replicating this methodology with a larger sample size, a sham-controlled group, more α-rTMS sessions, and electrophysiological measures for sensory sensitivities should be considered. The recruitment of medication and therapy-naïve participants may be required to accurately estimate the efficacy of α-rTMS.

## Supplementary Material

Figure_1_CONSORT_diagram_showing_the_flow_of_participants_through_the_trial_zpaf088

## Data Availability

Mediated access to non-identifiable study data is available from the corresponding author on written approval from funders to further protect the privacy and sensitivity of the health information of child participants.
