# Supplementary figures and images for: Evaluating the effect of repetitive transcranial magnetic stimulation on sleep difficulties in children with autism spectrum disorder: a randomized controlled trial
Source: Sleep Adv. 2025 Dec 5;6(4):zpaf088. doi: 10.1093/sleepadvances/zpaf088 (PMC12724088; doi:10.1093/sleepadvances/zpaf088)

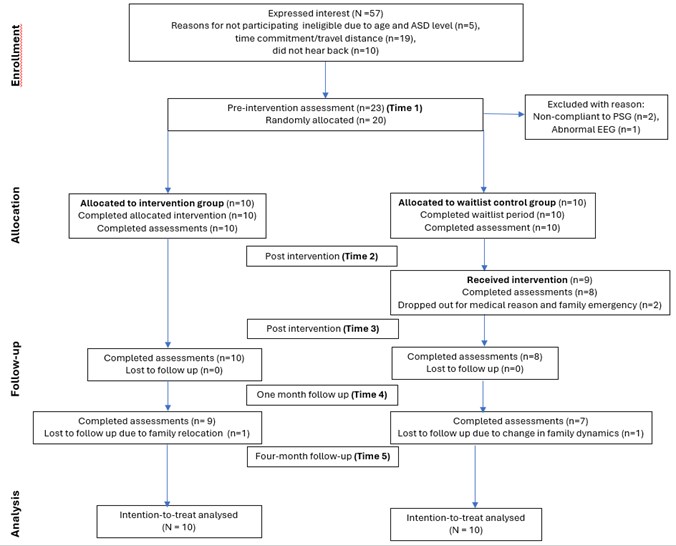

Supplement: Figure_1_CONSORT_diagram_showing_the_flow_of_participants_through_the_trial_zpaf088 [file figure_1_consort_diagram_showing_the_flow_of_participants_through_the_trial_zpaf088.jpeg]
